# 2-Amino-3-nitro­pyridinium 4-hy­droxy­benzene­sulfonate

**DOI:** 10.1107/S1600536812027651

**Published:** 2012-06-23

**Authors:** Yao-hui Lv, Wei Zhang, Hong Liu

**Affiliations:** a School of Materials Science and Engineering, Anhui Key Laboratory of Metal Materials and Processing, Anhui University of Technology, Anhui, Maanshan 243002, People’s Republic of China; b State Key Laboratory of Crystal Materials, Shandong University, Jinan 250100, People’s Republic of China

## Abstract

In the crystal structure of the title salt, C_5_H_6_N_3_O_2_^+^·C_6_H_5_O_4_S^−^, N—H⋯O and O—H⋯O hydrogen bonds link the cations and anions. The dihedral angle between the rings of the cation and anion is 79.91 (6)°.

## Related literature

For related structures, see: Nicoud *et al.* (1997 ▶); Akriche & Rzaigui (2009**a* ▶,b* ▶); Toumi Akriche *et al.* (2010 ▶); Koshima *et al.* (2004 ▶). For the design of second-order non-linear optical materials, see: Fur *et al.* (1998 ▶); Aakeröy *et al.* (1998 ▶). For information on the determination of non-linear optical properties, see: Kurtz & Perry (1968 ▶).
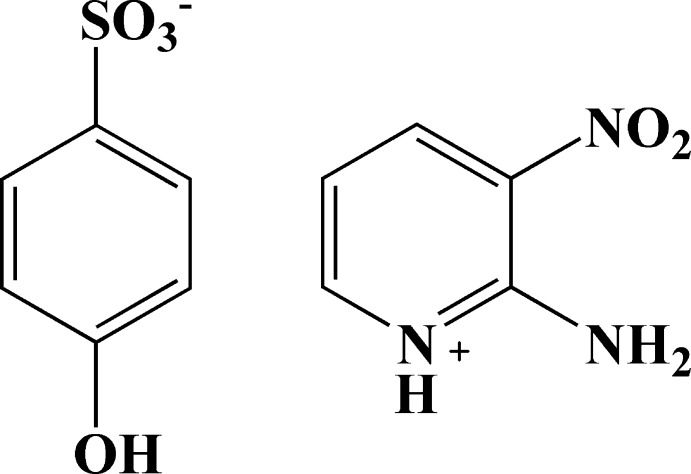


## Experimental

### 

#### Crystal data

C_5_H_6_N_3_O_2_^+^·C_6_H_5_O_4_S^−^
*M* *_r_* = 313.29
Monoclinic, 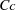

*a* = 9.0683 (19) Å
*b* = 13.5177 (16) Å
*c* = 10.9203 (17) Å
β = 94.042 (14)°
*V* = 1335.3 (4) Å^3^
*Z* = 4
Mo *K*α radiation
μ = 0.28 mm^−1^
*T* = 293 K
0.48 × 0.31 × 0.22 mm

#### Data collection

Rigaku Mercury CCD diffractometer
Absorption correction: multi-scan (*CrystalClear*; Rigaku, 2005 ▶) *T*_min_ = 0.879, *T*_max_ = 0.942
4067 measured reflections
2005 independent reflections
1989 reflections with *I* > 2σ(*I*)
*R*_int_ = 0.011

#### Refinement

*R*[*F* ^2^ > 2σ(*F* ^2^)] = 0.023
*wR*(*F* ^2^) = 0.063
*S* = 1.07
2005 reflections
194 parameters
2 restraints
H atoms treated by a mixture of independent and constrained refinement
Δρ_max_ = 0.12 e Å^−3^
Δρ_min_ = −0.20 e Å^−3^
Absolute structure: Flack (1983 ▶), 825 Friedel pairs
Flack parameter: 0.05 (6)

### 

Data collection: *CrystalClear* (Rigaku, 2005 ▶); cell refinement: *CrystalClear*; data reduction: *CrystalClear*; program(s) used to solve structure: *SHELXS97* (Sheldrick, 2008 ▶); program(s) used to refine structure: *SHELXL97* (Sheldrick, 2008 ▶); molecular graphics: *SHELXTL* (Sheldrick, 2008 ▶); software used to prepare material for publication: *SHELXL97*.

## Supplementary Material

Crystal structure: contains datablock(s) I, global. DOI: 10.1107/S1600536812027651/pk2420sup1.cif

Structure factors: contains datablock(s) I. DOI: 10.1107/S1600536812027651/pk2420Isup2.hkl

Supplementary material file. DOI: 10.1107/S1600536812027651/pk2420Isup3.cml

Additional supplementary materials:  crystallographic information; 3D view; checkCIF report

## Figures and Tables

**Table 1:** Hydrogen-bond geometry (Å, °)

*D*—H⋯*A*	*D*—H	H⋯*A*	*D*⋯*A*	*D*—H⋯*A*
N1—H1*A*⋯O5	0.86	2.01	2.862 (2)	172
N1—H1*B*⋯O1	0.86	2.08	2.674 (2)	126
N3—H3*A*⋯O6	0.86	1.88	2.734 (2)	170
N1—H1*B*⋯O3^i^	0.86	2.57	3.125 (3)	123
O3—H3⋯O4^ii^	0.75 (3)	1.98 (3)	2.688 (2)	158 (3)

Symmetry codes: (i) 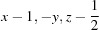; (ii) 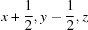.

**Fig. 1.**
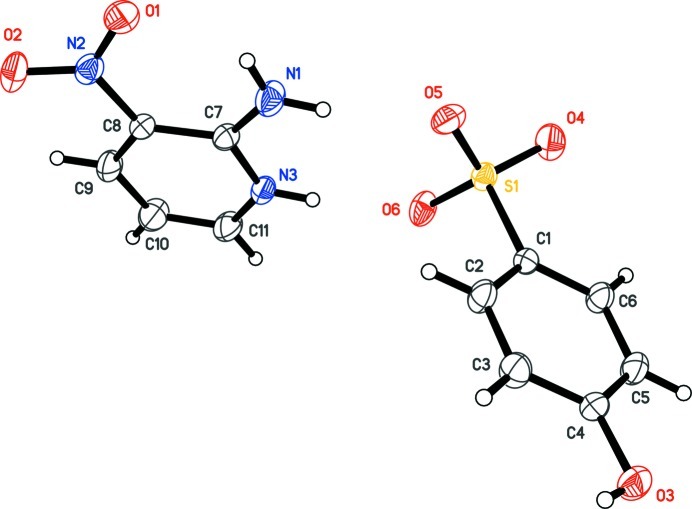
Molecular structure of the title compound. Non-hydrogen atoms are shown with the displacement ellipsoids at the 30% probability level.

**Fig. 2.**
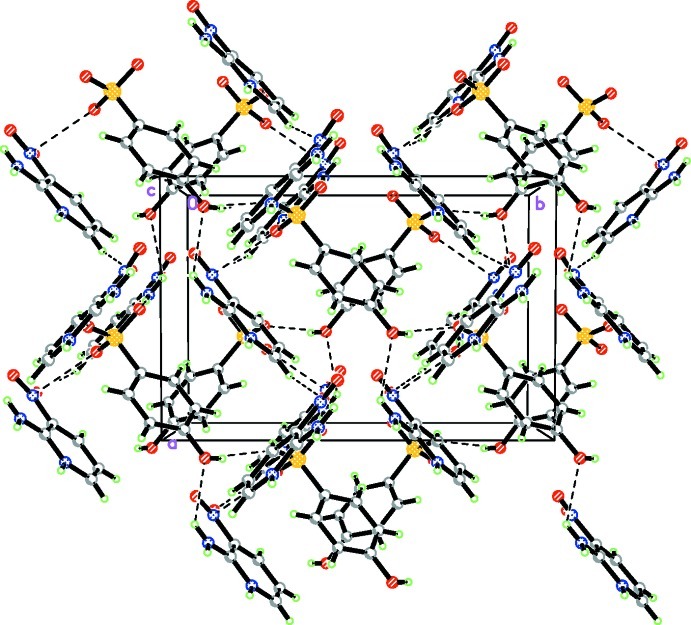
Crystal structure of the title compound viewed along the c axis, showing hydrogen-bonding associations as dashed lines.

## supplementary crystallographic information

### Comment

Research on new materials with large nonlinear optical (NLO) efficiencies has been extensively developed in the past two decades. A promising crystal-engineering strategy is to design organic cocrystals, using molecules with large macroscopic susceptibility components (Nicoud *et al.*, 1997). As an excellent donor-acceptor system with high susceptibility, 2-amino-3-nitropyridine cation has two electron-accepting centres, namely the NH_2_ amino-group and NH^+^ group in the pyridinium ring (Aakeröy *et al.*, 1998). Therefore, it is possible to utilize the cation of 2- amino-3-nitropyridinium as a nonlinear optical component to assemble potential NLO materials. Though several crystal structures based on the 2-amino-3-nitropyridine cation had been reported (Akriche & Rzaigui, 2009**a*,b*; Toumi Akriche *et al.*, 2010), to the best of our knowledge, the title complex in the present work is the first example of a NLO crystal with an efficiency as large as ten times that of the KDP standard (Kurtz & Perry, 1968). The asymmetric unit contains one anion and one cation that are shown in Fig. 1. Hydrogen bonding interactions, which construct a three-dimensional network, are listed in table 1. The crystal structure is stabilized by several hydrogen-bonding interactions formed within the crystal structure. These interactions link the cations and anions together in a complex spatial geometry, displayed in Fig. 2.

### Experimental

The title complex was synthesized from the mixture of 2-amino-3-nitropyridine with stoichiometric 4-hydroxybenzenesulfonic acid in ethanol solution. The reaction mixture was stirred for four hours and slowly heated to 45°C yielding a clear solution. After solvent evaporation at controlled temperature for several days, yellow block-shaped crystals were obtained in 90% yield. For second-harmonic generation (SHG) experiments (Kurtz and Perry, 1968), the polycrystalline samples were ground into powder and sieved using a series of mesh sizes in the range of 74–100 µm, and the SHG intensity was compared with KDP crystal.

### Refinement

All the H atoms bound to carbon and nitrogen were placed at idealized positions with respective bond lengths of C—H = 0.93 to 0.97 Å and N—H = 0.89 Å. and allowed to ride on their parent atoms with *U*_iso_ fixed at 1.2 *U*_eq_(C, *N*). The O-bound H atom was located in a difference Fourier map and refined isotropically.

### Crystal data

**Table:**

C_5_H_6_N_3_O_2_^+^·C_6_H_5_O_4_S^−^	2-Amino-3-nitropyridine 4-hydroxybenzenesulfonate
*M**_r_* = 313.29	*D*_x_ = 1.558 Mg m^−^^3^
Monoclinic, *C**c*	Melting point: 481 K
Hall symbol: C -2yc	Mo *K*α radiation, λ = 0.71073 Å
*a* = 9.0683 (19) Å	Cell parameters from 1991 reflections
*b* = 13.5177 (16) Å	θ = 3.0–27.5°
*c* = 10.9203 (17) Å	µ = 0.28 mm^−^^1^
β = 94.042 (14)°	*T* = 293 K
*V* = 1335.3 (4) Å^3^	Block, yellow
*Z* = 4	0.48 × 0.31 × 0.22 mm
*F*(000) = 648	

### Data collection

**Table:**

Rigaku Mercury CCD diffractometer	2005 independent reflections
Radiation source: fine-focus sealed tube	1989 reflections with *I* > 2σ(*I*)
Graphite monochromator	*R*_int_ = 0.011
ω and φ scans'	θ_max_ = 25.0°, θ_min_ = 2.7°
Absorption correction: multi-scan (*CrystalClear*; Rigaku, 2005)	*h* = −10→10
*T*_min_ = 0.879, *T*_max_ = 0.942	*k* = −16→16
4067 measured reflections	*l* = −12→12

### Refinement

**Table:**

Refinement on *F*^2^	Secondary atom site location: difference Fourier map
Least-squares matrix: full	Hydrogen site location: inferred from neighbouring sites
*R*[*F*^2^ > 2σ(*F*^2^)] = 0.023	H atoms treated by a mixture of independent and constrained refinement
*wR*(*F*^2^) = 0.063	*w* = 1/[σ^2^(*F*_o_^2^) + (0.0386*P*)^2^ + 0.3606*P*] where *P* = (*F*_o_^2^ + 2*F*_c_^2^)/3
*S* = 1.07	(Δ/σ)_max_ < 0.001
2005 reflections	Δρ_max_ = 0.12 e Å^−^^3^
194 parameters	Δρ_min_ = −0.20 e Å^−^^3^
2 restraints	Absolute structure: Flack (1983), 825 Friedel pairs
Primary atom site location: structure-invariant direct methods	Flack parameter: 0.05 (6)

### Special details

**Table:**

Geometry. All e.s.d.'s (except the e.s.d. in the dihedral angle between two l.s. planes) are estimated using the full covariance matrix. The cell e.s.d.'s are taken into account individually in the estimation of e.s.d.'s in distances, angles and torsion angles; correlations between e.s.d.'s in cell parameters are only used when they are defined by crystal symmetry. An approximate (isotropic) treatment of cell e.s.d.'s is used for estimating e.s.d.'s involving l.s. planes.
Refinement. Refinement of *F*^2^ against ALL reflections. The weighted *R*-factor *wR* and goodness of fit *S* are based on *F*^2^, conventional *R*-factors *R* are based on *F*, with *F* set to zero for negative *F*^2^. The threshold expression of *F*^2^ > σ(*F*^2^) is used only for calculating *R*-factors(gt) *etc*. and is not relevant to the choice of reflections for refinement. *R*-factors based on *F*^2^ are statistically about twice as large as those based on *F*, and *R*- factors based on ALL data will be even larger.

### Fractional atomic coordinates and isotropic or equivalent isotropic displacement parameters (Å^2^)

**Table:**

	*x*	*y*	*z*	*U*_iso_*/*U*_eq_	
S1	0.61553 (5)	0.16714 (3)	0.00229 (4)	0.03441 (13)	
O1	0.27111 (18)	0.05257 (12)	−0.51298 (15)	0.0525 (4)	
O2	0.35055 (19)	0.09208 (11)	−0.68818 (14)	0.0566 (4)	
O3	1.09361 (18)	−0.09220 (13)	0.20630 (15)	0.0517 (4)	
O4	0.57072 (19)	0.22726 (11)	0.10356 (14)	0.0554 (5)	
O5	0.49921 (15)	0.10255 (11)	−0.04791 (14)	0.0475 (4)	
O6	0.67629 (17)	0.22667 (11)	−0.09397 (13)	0.0468 (4)	
N1	0.4364 (2)	0.09853 (13)	−0.30840 (16)	0.0513 (5)	
H1A	0.4637	0.1008	−0.2314	0.062*	
H1B	0.3651	0.0603	−0.3342	0.062*	
N2	0.35713 (19)	0.09660 (12)	−0.57633 (16)	0.0417 (4)	
N3	0.61555 (19)	0.21303 (12)	−0.34220 (15)	0.0407 (4)	
H3A	0.6404	0.2109	−0.2648	0.049*	
C3	0.9047 (3)	−0.05629 (15)	0.0470 (2)	0.0453 (5)	
H1C	0.9272	−0.1133	0.0045	0.054*	
C4	0.9832 (2)	−0.03311 (14)	0.15597 (17)	0.0369 (4)	
C10	0.6574 (3)	0.28190 (19)	−0.5342 (2)	0.0583 (6)	
H3B	0.7071	0.3261	−0.5819	0.070*	
C6	0.8393 (2)	0.11399 (14)	0.17267 (17)	0.0400 (5)	
H4A	0.8172	0.1712	0.2149	0.048*	
C11	0.6903 (3)	0.27529 (18)	−0.4120 (2)	0.0514 (6)	
H5A	0.7657	0.3142	−0.3753	0.062*	
C9	0.5476 (3)	0.22104 (17)	−0.58661 (19)	0.0485 (5)	
H6A	0.5247	0.2231	−0.6709	0.058*	
C2	0.7930 (2)	0.00511 (15)	0.00116 (18)	0.0417 (5)	
H7A	0.7403	−0.0106	−0.0724	0.050*	
C7	0.5037 (2)	0.15357 (14)	−0.38655 (17)	0.0356 (4)	
C8	0.4725 (2)	0.15784 (13)	−0.51561 (17)	0.0348 (4)	
C5	0.9519 (2)	0.05312 (16)	0.21828 (18)	0.0451 (5)	
H10A	1.0069	0.0697	0.2905	0.054*	
C1	0.7585 (2)	0.09035 (13)	0.06404 (17)	0.0303 (4)	
H3	1.099 (3)	−0.136 (2)	0.164 (3)	0.073 (10)*	

### Atomic displacement parameters (Å^2^)

**Table:**

	*U*^11^	*U*^22^	*U*^33^	*U*^12^	*U*^13^	*U*^23^
S1	0.0370 (3)	0.0340 (2)	0.0317 (2)	0.0036 (2)	−0.00145 (17)	0.00220 (19)
O1	0.0457 (9)	0.0554 (9)	0.0561 (10)	−0.0104 (7)	0.0020 (8)	−0.0067 (8)
O2	0.0704 (12)	0.0594 (9)	0.0378 (8)	0.0024 (8)	−0.0123 (8)	−0.0111 (7)
O3	0.0527 (10)	0.0561 (9)	0.0451 (8)	0.0195 (7)	−0.0039 (7)	0.0041 (7)
O4	0.0701 (12)	0.0529 (9)	0.0430 (9)	0.0280 (8)	0.0026 (8)	−0.0038 (7)
O5	0.0361 (8)	0.0538 (8)	0.0510 (9)	−0.0047 (6)	−0.0078 (7)	0.0087 (7)
O6	0.0572 (10)	0.0455 (8)	0.0361 (8)	−0.0100 (7)	−0.0073 (7)	0.0109 (6)
N1	0.0538 (11)	0.0628 (11)	0.0362 (9)	−0.0152 (9)	−0.0041 (8)	0.0116 (8)
N2	0.0412 (10)	0.0407 (9)	0.0418 (10)	0.0083 (7)	−0.0063 (8)	−0.0073 (7)
N3	0.0378 (10)	0.0541 (9)	0.0295 (8)	−0.0040 (7)	−0.0038 (7)	0.0022 (7)
C3	0.0538 (14)	0.0403 (10)	0.0411 (11)	0.0096 (9)	−0.0008 (10)	−0.0083 (9)
C4	0.0339 (11)	0.0417 (9)	0.0350 (10)	0.0063 (8)	0.0006 (8)	0.0049 (8)
C10	0.0507 (14)	0.0799 (16)	0.0439 (13)	−0.0221 (12)	0.0014 (10)	0.0151 (11)
C6	0.0438 (12)	0.0407 (10)	0.0345 (10)	0.0071 (8)	−0.0045 (9)	−0.0081 (8)
C11	0.0420 (12)	0.0671 (14)	0.0444 (12)	−0.0167 (11)	−0.0025 (10)	0.0023 (10)
C9	0.0490 (13)	0.0655 (14)	0.0304 (11)	−0.0046 (11)	−0.0022 (10)	0.0068 (10)
C2	0.0472 (12)	0.0412 (10)	0.0352 (10)	0.0068 (8)	−0.0081 (9)	−0.0083 (8)
C7	0.0335 (11)	0.0386 (9)	0.0341 (10)	0.0037 (7)	−0.0020 (8)	0.0016 (8)
C8	0.0303 (11)	0.0424 (9)	0.0312 (9)	0.0028 (7)	−0.0017 (8)	0.0002 (7)
C5	0.0461 (13)	0.0551 (11)	0.0326 (10)	0.0060 (9)	−0.0067 (9)	−0.0069 (9)
C1	0.0305 (10)	0.0322 (8)	0.0281 (9)	0.0021 (7)	0.0019 (7)	0.0017 (7)

### Geometric parameters (Å, º)

**Table:**

S1—O5	1.4470 (15)	C3—C4	1.379 (3)
S1—O4	1.4532 (15)	C3—H1C	0.9300
S1—O6	1.4622 (15)	C4—C5	1.389 (3)
S1—C1	1.7580 (19)	C10—C11	1.350 (3)
O1—N2	1.232 (2)	C10—C9	1.384 (3)
O2—N2	1.220 (2)	C10—H3B	0.9300
O3—C4	1.365 (2)	C6—C5	1.378 (3)
O3—H3	0.75 (3)	C6—C1	1.387 (3)
N1—C7	1.314 (2)	C6—H4A	0.9300
N1—H1A	0.8600	C11—H5A	0.9300
N1—H1B	0.8601	C9—C8	1.368 (3)
N2—C8	1.456 (2)	C9—H6A	0.9300
N3—C11	1.350 (3)	C2—C1	1.388 (3)
N3—C7	1.357 (2)	C2—H7A	0.9300
N3—H3A	0.8600	C7—C8	1.419 (3)
C3—C2	1.376 (3)	C5—H10A	0.9300
			
O5—S1—O4	112.97 (10)	C5—C6—C1	120.34 (18)
O5—S1—O6	111.18 (8)	C5—C6—H4A	119.8
O4—S1—O6	112.28 (8)	C1—C6—H4A	119.8
O5—S1—C1	106.70 (8)	N3—C11—C10	121.0 (2)
O4—S1—C1	106.05 (9)	N3—C11—H5A	119.5
O6—S1—C1	107.19 (9)	C10—C11—H5A	119.5
C4—O3—H3	107 (2)	C8—C9—C10	120.65 (19)
C7—N1—H1A	120.0	C8—C9—H6A	119.7
C7—N1—H1B	120.0	C10—C9—H6A	119.7
H1A—N1—H1B	120.0	C3—C2—C1	120.45 (17)
O2—N2—O1	123.34 (18)	C3—C2—H7A	119.8
O2—N2—C8	117.85 (19)	C1—C2—H7A	119.8
O1—N2—C8	118.80 (16)	N1—C7—N3	118.24 (16)
C11—N3—C7	124.10 (18)	N1—C7—C8	126.78 (17)
C11—N3—H3A	117.9	N3—C7—C8	114.98 (17)
C7—N3—H3A	117.9	C9—C8—C7	121.06 (18)
C2—C3—C4	119.90 (18)	C9—C8—N2	117.87 (17)
C2—C3—H1C	120.1	C7—C8—N2	121.05 (17)
C4—C3—H1C	120.1	C6—C5—C4	119.74 (17)
O3—C4—C3	122.25 (18)	C6—C5—H10A	120.1
O3—C4—C5	117.58 (18)	C4—C5—H10A	120.1
C3—C4—C5	120.17 (17)	C6—C1—C2	119.37 (17)
C11—C10—C9	118.2 (2)	C6—C1—S1	121.52 (15)
C11—C10—H3B	120.9	C2—C1—S1	119.10 (14)
C9—C10—H3B	120.9		
			
C2—C3—C4—O3	179.0 (2)	O2—N2—C8—C7	−168.96 (18)
C2—C3—C4—C5	−1.4 (3)	O1—N2—C8—C7	11.4 (3)
C7—N3—C11—C10	−0.3 (4)	C1—C6—C5—C4	−1.0 (3)
C9—C10—C11—N3	−1.8 (4)	O3—C4—C5—C6	−178.49 (19)
C11—C10—C9—C8	1.6 (4)	C3—C4—C5—C6	1.9 (3)
C4—C3—C2—C1	−0.1 (3)	C5—C6—C1—C2	−0.5 (3)
C11—N3—C7—N1	−177.9 (2)	C5—C6—C1—S1	−179.21 (16)
C11—N3—C7—C8	2.4 (3)	C3—C2—C1—C6	1.0 (3)
C10—C9—C8—C7	0.6 (3)	C3—C2—C1—S1	179.78 (18)
C10—C9—C8—N2	179.3 (2)	O5—S1—C1—C6	−141.59 (17)
N1—C7—C8—C9	177.7 (2)	O4—S1—C1—C6	−20.9 (2)
N3—C7—C8—C9	−2.6 (3)	O6—S1—C1—C6	99.23 (19)
N1—C7—C8—N2	−0.9 (3)	O5—S1—C1—C2	39.69 (19)
N3—C7—C8—N2	178.80 (16)	O4—S1—C1—C2	160.38 (17)
O2—N2—C8—C9	12.3 (3)	O6—S1—C1—C2	−79.49 (18)
O1—N2—C8—C9	−167.30 (19)		

### Hydrogen-bond geometry (Å, º)

**Table:**

*D*—H···*A*	*D*—H	H···*A*	*D*···*A*	*D*—H···*A*
N1—H1*A*···O5	0.86	2.01	2.862 (2)	172
N1—H1*B*···O1	0.86	2.08	2.674 (2)	126
N3—H3*A*···O6	0.86	1.88	2.734 (2)	170
N1—H1*B*···O3^i^	0.86	2.57	3.125 (3)	123
O3—H3···O4^ii^	0.75 (3)	1.98 (3)	2.688 (2)	158 (3)

Symmetry codes: (i) *x*−1, −*y*, *z*−1/2; (ii) *x*+1/2, *y*−1/2, *z*.
